# Efficacy, safety, and prognostic factors of PD-1 inhibitors combined with lenvatinib and Gemox chemotherapy as first-line treatment in advanced intrahepatic cholangiocarcinoma: a multicenter real-world study

**DOI:** 10.1007/s00262-023-03466-8

**Published:** 2023-05-29

**Authors:** Chengpei Zhu, Hu Li, Xiaobo Yang, Shanshan Wang, Yunchao Wang, Nan Zhang, Yanyu Wang, Jingnan Xue, Longhao Zhang, Cong Ning, Xu Yang, Ziyu Xun, Jiashuo Chao, Junyu Long, Xinting Sang, Zhenyu Zhu, Haitao Zhao

**Affiliations:** 1 Department of Liver Surgery, State Key Laboratory of Complex Severe and Rare Diseases, Peking Union Medical College Hospital, Chinese Academy of Medical Sciences and Peking Union Medical College (CAMS & PUMC), #1 Shuaifuyuan, Wangfujing, Beijing, 100730 China; 2 Department of Hepatobiliary Surgery, The Fifth Medical Center of PLA General Hospital, No. 100 Middle Road of West 4th Ring Road, Fengtai District, Beijing, 100039 China

**Keywords:** Advanced intrahepatic cholangiocarcinoma, Lenvatinib, PD-1 inhibitor, Gemox chemotherapy, Systemic therapy

## Abstract

**Background:**

A programmed cell death protein-1 (PD-1) inhibitor combined with lenvatinib and Gemox chemotherapy as first-line therapy demonstrated high anti-tumor activity against biliary tract cancer in phase II clinical trials. Herein, we aimed to investigate the efficacy and safety for advanced intrahepatic cholangiocarcinoma (ICC) in a multicenter real-world study.

**Methods:**

Patients with advanced ICC who received PD-1 inhibitor combined with lenvatinib and Gemox chemotherapy were retrospectively screened at two medical centers. The primary endpoints were overall survival (OS) and progression-free survival (PFS), whereas the secondary endpoints were objective response rate (ORR), disease control rate (DCR), and safety. Prognostic factors for survival were analyzed.

**Results:**

Fifty-three patients with advanced ICC were included in this study. The median follow-up time was 13.7 (95% confidence interval (CI): 12.9–17.2) months. The median OS and PFS were 14.3 (95% CI: 11.3–NR) and 8.63 (95% CI: 7.17–11.6) months, respectively. The ORR, DCR, and clinical benefit rate were 52.8, 94.3, and 75.5%, respectively. In the multivariate analysis, the tumor burden score (TBS), tumor-node metastasis classification (TNM) stage, and PD-L1 expression were independent prognostic factors for OS and PFS. All patients experienced adverse events (AEs), 41.5% (22/53) experienced grade 3 or 4 AEs, including fatigue (8/53, 15.1%) and myelosuppression (7/53, 13.2%). No grade 5 AEs were reported.

**Conclusion:**

PD-1 inhibitors combined with lenvatinib and Gemox chemotherapy represent an effective and tolerable regimen for advanced ICC in a multicenter retrospective real-world study. TBS, TNM stage, and PD-L1 expression can be used as potential prognostic factors for OS and PFS.

**Supplementary Information:**

The online version contains supplementary material available at 10.1007/s00262-023-03466-8.

## Introduction

Intrahepatic cholangiocarcinoma (ICC) is one of the most common biliary tract cancers (BTC), with hidden onset, high malignancy, strong invasion, and poor prognosis [1, 2]. Surgical resection is the best treatment option for patients with early ICC. However, early ICC diagnosis lacks sufficient specificity and sensitivity. Systemic therapy is the primary treatment option for advanced ICC. Gemcitabine, platinum, fluorouracil, and albumin-bound paclitaxel are the main chemotherapeutic agents used as the first-line chemotherapy for advanced BTC. The ABC-002 study established gemcitabine plus cisplatin (GC) as the standard first-line treatment, with a median overall survival (OS) and progression-free survival (PFS) of 11.7 and 8.0 months in the GC group, respectively [3]. The ABC-06 study confirmed that folinic acid, fluorouracil, and oxaliplatin (FOLFOX) chemotherapy could be used as a second-line regimen for BTC, and the median OS of patients in the FOLFOX chemotherapy group was prolonged (6.2 vs. 5.3 months) compared with the active symptom control group [4]. Generally, the overall effect of chemotherapy is limited, and once patients develop resistance or disease progression, the treatment options are limited.

With continuous research on immune checkpoint inhibitors (ICIs), targeted therapy, and related combination therapy, more options have been provided for the treatment of advanced BTC, including ICC [5–10]. Drug resistance is one of the reasons that limit the efficacy of antitumor treatments. Combining drugs with different mechanisms of action may help overcome multiple drug resistance mechanisms. Most chemotherapeutic agents act through their direct cytotoxic effects without considering their impact on the immune system, and chemotherapy-resistant patients respond to chemotherapy rechallenge after anti-PD-1 therapy [11]. Chemotherapy can increase the response to immunotherapy by increasing the immunogenicity of tumor cells or inhibiting the immunosuppressive circuit [12, 13]. Lenvatinib is a multi-targeted tyrosine kinase inhibitor that targets vascular endothelial growth factor receptors (VEGFR1-3) and participates in the immune response by playing a role in the VEGF-VEGFR pathway [14, 15], suggesting that combination treatment with different mechanisms may play a promising role in advanced ICC.

A phase II study of tislelizumab, a PD-1 inhibitor, combined with lenvatinib, oxaliplatin, and gemcitabine (Gemox) chemotherapy used as first-line treatment for potentially resectable locally advanced BTC showed an objective response rate (ORR) of 56% and a conversion surgical resection rate of 52% [16]. Another phase II clinical trial suggested that lenvatinib combined with toripalimab, a PD-1 inhibitor, plus Gemox chemotherapy as first-line therapy, showed good efficacy in advanced ICC, with an ORR of 80% and a median PFS of 10.0 months [17]. These studies suggest that triple therapy (PD-1 inhibitors combined with lenvatinib and Gemox chemotherapy) have good efficacy for BTC. Recently, our team demonstrated the role of triple therapy in advanced BTC in a real-world study, which showing an ORR of 43.9%, median OS of 13.4 months, and median PFS of 9.27 months [18]. However, the study was only a single-center study, included both first-line treatment and non-first-line treatment for BTC patients, and fewer ICC patients were treated with triple therapy as the first-line treatment (only 14 cases) [18]. These low sample size data cannot provide a detailed understanding of the exact efficacy of triple therapy as the first-line treatment for advanced ICC in a real world study.

Based on the above research results, we conducted a multicenter retrospective study to evaluate the efficacy, safety, and prognostic factors for survival of PD-1 inhibitors combined with lenvatinib and Gemox chemotherapy as first-line systemic therapy for patients with advanced ICC in a real-world study. We believe that PD-1 inhibitors plus lenvatinib and Gemox chemotherapy may be an exciting therapeutic regimen for patients with advanced ICC.

## Materials and methods

### Study design and population

**Fig. 1:**
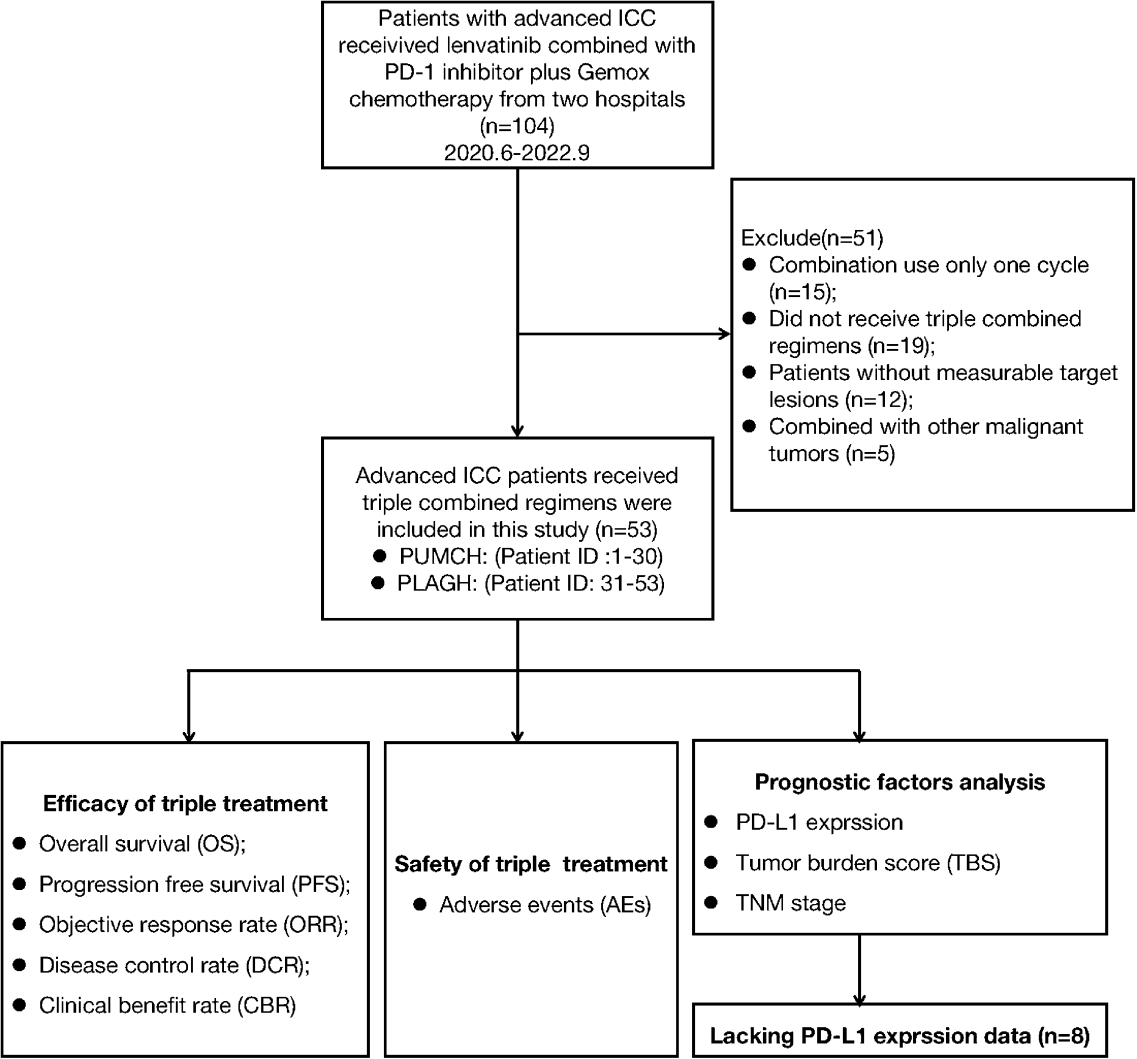
Flow diagram of the study population

This multicenter retrospective study assessed the efficacy and safety of PD-1 inhibitors combined with lenvatinib plus Gemox chemotherapy as first-line therapy for advanced ICC at the Peking Union Medical College Hospital (PUMCH) and The Fifth Medical Center of PLA General Hospital (PLAGH). A total of 104 patients with advanced ICC who received a PD-1 inhibitor combined with lenvatinib and Gemox chemotherapy were enrolled in this study from June 2020 to September 2022. The primary eligibility criteria were histologically confirmed intrahepatic cholangiocarcinoma and at least one measurable tumor lesion according to the RECIST v1.1 criteria [19]. Among the initial 104 patients, 15 had combined use of one cycle, 19 did not receive triple combined regimens, 12 did not have measurable target lesions, and 5 had other additional malignant tumors (Fig. 1). Finally, 53 patients were enrolled in this study, 30 and 23 were enrolled in the PUMCH and PLAGH groups, respectively. Information on age, sex, Eastern Cooperative Oncology Group (ECOG) performance status, Child–Pugh score, carbohydrate antigen 19-9 (CA19-9), carcinoembryonic antigen (CEA), hepatitis B virus (HBV) infection, maximum tumor diameter, tumor burden score (TBS), differentiated histology, tumor-node metastasis classification (TNM) stage, site of metastases, PD-L1 expression, and type of PD-1 inhibitors were compiled and recorded (Table 1). The TBS was calculated based on the maximum tumor size and the number of tumors in the liver [20, 21].

**Table 1:** Baseline characteristics of the study population

Parameters	Total (n = 53)
*Age, years *(*median, IQR*)	58 (51–66)
≥ 60	22 [41.5%]
< 60	31 [58.5%]
*Sex, n *[*%*]	
Female	20 [37.7]
Male	33 [62.3]
*ECOG performance status, n *[*%*]	
0	28 [52.8]
1	23 [43.4]
2	2 [3.8]
*Child–Pugh score, n *[*%*]	
A	32 [60.4]
B	21 [39.6]
*CA19-9, U/mL *(*median, IQR*)	210 (35.8–1398)
≥ 200	30 [56.6%]
< 200	23 [43.4%]
*CEA, ng/ml *(*median, IQR*)	4.2 (2.5–13.9)
≥ 5	22 [41.5%]
< 5	31 [58.5%]
HBV infection, n [%]	10 [18.9]
*Maximum tumor diameter, cm *(*median, IQR*)	5.4 (3.6–7.6)
≥ 5	29 [54.7%]
< 5	24 [45.3%]
*TBS, n *[*%*]	
≥ 8	22 [41.5]
< 8	31 [58.5]
*Differentiated histology, n *[*%*]	
Poor	17 [32.1]
Moderate	26 [49.1]
Well	5 [9.4]
NA	5 [9.4]
*TNM stage, n *[*%*]	
III	23 [43.4]
IV	30 [56.6]
*Site of metastases, n *[*%*]	
Intrahepatic	36 [67.9]
Lymph nodes	34 [64.2]
Lung	7 [13.2]
Bone	4 [7.5]
Others	5 [9.4]
*PD-L1 expression, n *[*%*]	
Positive	17 [32.1]
Negative	28 [52.8]
NA	8 [15.1]
*Type of PD-1 inhibitors, n [%]*	
Toripalimab	29 [54.7]
Tislelizumab	11 [20.8]
Camrelizumab	7 [13.2]
Pembrolizumab	6 [11.3]

*ECOG* Eastern Cooperative Oncology Group, *CA19-9* carbohydrate antigen 19-9, *CEA* carcinoembryonic antigen, *HBV* hepatitis type B virus, *TBS* tumor burden score, *TNM* tumor node metastasis classification, *PD-L1* programmed cell death ligand 1, *PD-1* programmed cell death 1

### Treatment protocol

Information regarding the dates of initiation and completion of treatment, initial dose, radiological evaluation, laboratory data, and adverse events (AEs) during treatment was systematically collected. Lenvatinib was administered orally at a dose of 12 mg (for patients with body weight ≥ 60 kg) or 8 mg (for patients with body weight < 60 kg) once a day. Anti-PD-1 antibodies were administered at a fixed dose of 200 mg (240 mg for toripalimab) or a fixed dose of 3 mg/kg body weight every 3 weeks. The Gemox chemotherapy regimen was administered as 1 g/m^2^ of gemcitabine on days 1 and 8, 100 mg/m^2^ of oxaliplatin on day 1, and every 3 weeks by IV injection for six cycles.

### Response assessment and safety evaluation

The clinical objective response was measured using the RECIST v1.1 criteria [19] and evaluated by professional radiologists at PUMCH and PLAGH. Computed tomography (CT) and magnetic resonance imaging (MRI) were performed to assess treatment response. The primary endpoints were OS and PFS, whereas the secondary endpoints were the ORR, disease control rate (DCR), clinical benefit rate (CBR) and safety. CBR was defined as the proportion of patients with a radiologically confirmed objective response (complete response [CR] or partial response [PR]) or stable disease (SD) for > 6 months [22]. Safety were recorded by physical examination, laboratory evaluation, and electronic medical records or collected by the investigators using the Common Terminology Criteria for Adverse Events (version 5.0) as a reference [23].

### Evaluation of PD-L1 expression

Whole sections from formalin-fixed, paraffin-embedded tumor specimens were subjected to immunohistochemistry. For each tissue slice, 5-μm-thick sections were selected and placed on glass slides. The primary antibody used was anti-PD-L1, followed by the addition of secondary antibodies to all sections, including the negative control slides. Evaluation of PD-L1 expression was performed by independent pathologists who were blinded to the clinicopathological data, including therapeutic response and survival time. PD-L1 positivity or overexpression was defined as > 5% positive expression in tumor cells.

### Statistical analysis

The cutoff date for analysis was December 30, 2022 in this study. Survival curves were estimated using the Kaplan–Meier method, and the log-rank test were used to analyzed the comparison groups. Hazard ratios (HRs) of each clinical factors for PFS and OS were estimated using the Cox proportional hazard model. For comparisons of individual variables, the *t*-test, Mann–Whitney U test, χ^2^ test, and Fisher’s exact test were performed as appropriate. Results with two-tailed p-values < 0.05 were considered statistically significant. Statistical analyses were performed using R-4.2.0 (https://www.r-project.org/) and the SPSS 25 software.

## Results

### Baseline characteristics

We screened 104 patients with advanced ICC who were treated in PUMCH and PLAGH from June 2020 to September 2022; 51 patients were excluded from the study (Fig. 1). Finally, we included 53 patients with advanced ICC who received PD-1 inhibitors combined with lenvatinib and Gemox chemotherapy as first-line treatment. The demographic and baseline characteristics of the 53 patients are summarized in Table 1. At the time of initial treatment, the median age was 58 years, with 41.5% of the patients being over 60 years old and 37.7% being women. We observed that 28 (52.8%) patients had an ECOG performance status of 0, and 32 (60.4%) patients had Child–Pugh score A. At baseline, the median CA19-9 level was 210 U/mL; 56.6% of the patients had a level > 200 U/mL. The median CEA level was 4.2 ng/mL; 41.5% of the patients had a level > 5 ng/mL. We observed that 10 patients (18.9%) had a history of HBV infection. The median maximum tumor diameter was 5.4 cm, and 54.7% of the patients had a level > 5 cm. At baseline, 22 patients (41.5%) had TBS > 8. In total, 17 (32.1%) patients had poorly differentiated histology, 23 (43.4%) had TNM stage III, and 17 (32.1%) had positive PD-L1 expression. Further, we observed that before treatment, most patients had metastatic tumors in the liver (36/53, 67.9%), lymph nodes (34/53, 64.2%), lungs (7/53, 13.2%), and bones (4/53, 7.5%). Among the 53 patients who had received different types of PD-1 inhibitors, 29 (54.7%) were treated with the toripalimab regimen, 11 (20.8%) with the tislelizumab regimen, 7 (13.2%) with the camrelizumab regimen, and 6 (11.3%) with the pembrolizumab regimen.

### Treatment and efficacy

**Fig. 2:**
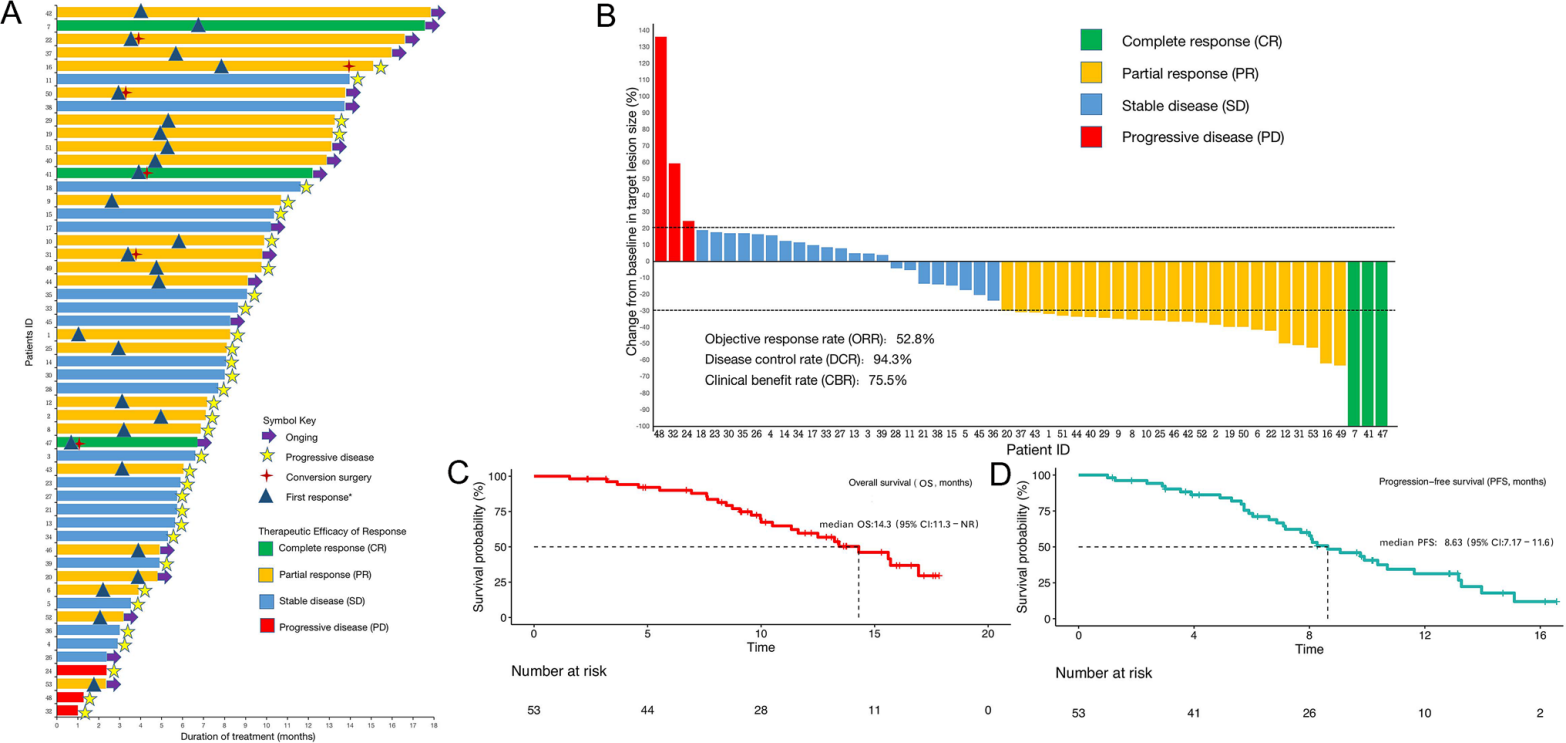
Therapeutic efficacy of PD-1 inhibitors combined with lenvatinib plus Gemox chemotherapy in patients with advanced intrahepatic cholangiocarcinoma. Treatment duration (**a**). Maximum percentage change in the sum of the diameters of the target lesions from baseline (**b**). Kaplan–Meier estimation of overall survival (**c**) and progression-free survival (**d**) in the entire cohort. *The first response was defined as the first time assessed as partial or complete response

The median duration of treatment with PD-1 inhibitors combined with lenvatinib and Gemox chemotherapy was 8.07 (interquartile range: 5.3–11.6) months. The treatment duration for all the patients is shown in Fig. 2a. The median follow-up time was 13.7 (95% confidence interval (CI): 12.9–17.2) months for all participants in our cohort. All patients underwent a complete radiological evaluation. Overall, we identified 36 (67.9%) patients with decreased tumor sizes from baseline (Fig. 2b). Interestingly, we observed that 28 (52.8%) patients achieved an objective response, including 3 (5.7%) who showed CR and 25 (47.1%) who showed PR. We observed that 22 (41.5%) patients exhibited SD, whereas 3 (5.7%) exhibited PD. Consistently, we observed that the overall radiologically confirmed ORR was 52.8% (95% CI: 39.7–65.6%), and DCR was 94.3% (95% CI: 84.6–98.1%) (Fig. 2b, Table 2).

**Table 2:** Therapeutic efficacy of response and survival outcomes

Therapeutic response assessment	Entire cohort (n = 53)
Objective response rate (ORR, n, %, 95% CI)	28, 52.8 (39.7–65.6)
Complete response (CR, n, %)	3 (5.7)
Partial response (PR, n, %)	25 (47.1)
Stable disease (SD, n, %)	22 (41.5)
Progressive disease (PD, n, %)	3 (5.7)
Disease control rate (DCR, n, %, 95% CI)	50, 94.3 (84.6–98.1)
Clinical benefit rate (CBR, n, %, 95% CI)	40, 75.5 (62.4–85.1)
Median progression free survival (mPFS, months, 95% CI)	8.63 (7.17–11.6)
6 months PFS (%, 95% CI)	73.3 (61.8–86.9)
12 months PFS (%, 95% CI)	31.3 (19.6–49.9)
Median overall survival (mOS, months, 95% CI)	14.3 (11.3–NR)
6 months OS (%, 95% CI)	90.0 (82.1–98.7)
12 months OS (%, 95% CI)	59.6 (46.6–76.3)

We investigated the survival outcomes of the enrolled patients. For the entire cohort, we observed that the median OS was 14.3 (95% CI: 11.3–NR) and the median PFS was 8.63 (95% CI: 7.17–11.6) months (Fig. 2c, d, Table 2). The 6 months and 12 months OS were 90.0% (95% CI: 82.1–98.7%) and 59.6% (95% CI: 46.6–76.3%), respectively (Table 2). The 6 months and 12 months PFS were 73.3% (95% CI: 61.8–86.9%) and 31.3% (95% CI: 19.6–49.9%), respectively (Table 2). We further determined CBR in all patients. We observed that the CBR in all 53 patients was 75.5% (95% CI: 62.4–85.1%) (Fig. 2b, Table 2).

Six patients underwent conversion surgery after triple combination treatment, with a patient ID of 16, 22, 31, 41, 47, and 50, respectively (Fig. 2a, Table S1). Four patients achieved PR, and two achieved CR before conversion surgery. A median of 5 cycles of triple therapy was administered before conversion surgery (Table S1). One patient (patient ID 16) had disease progression 1.2 months after undergoing conversion surgery and changed treatment regimens. The remaining 5 patients had no disease progression at the time of the last follow-up and were receiving maintenance therapy with a PD-1 inhibitor plus lenvatinib.

### Subgroup analyses and prognostic factors

**Fig. 3:**
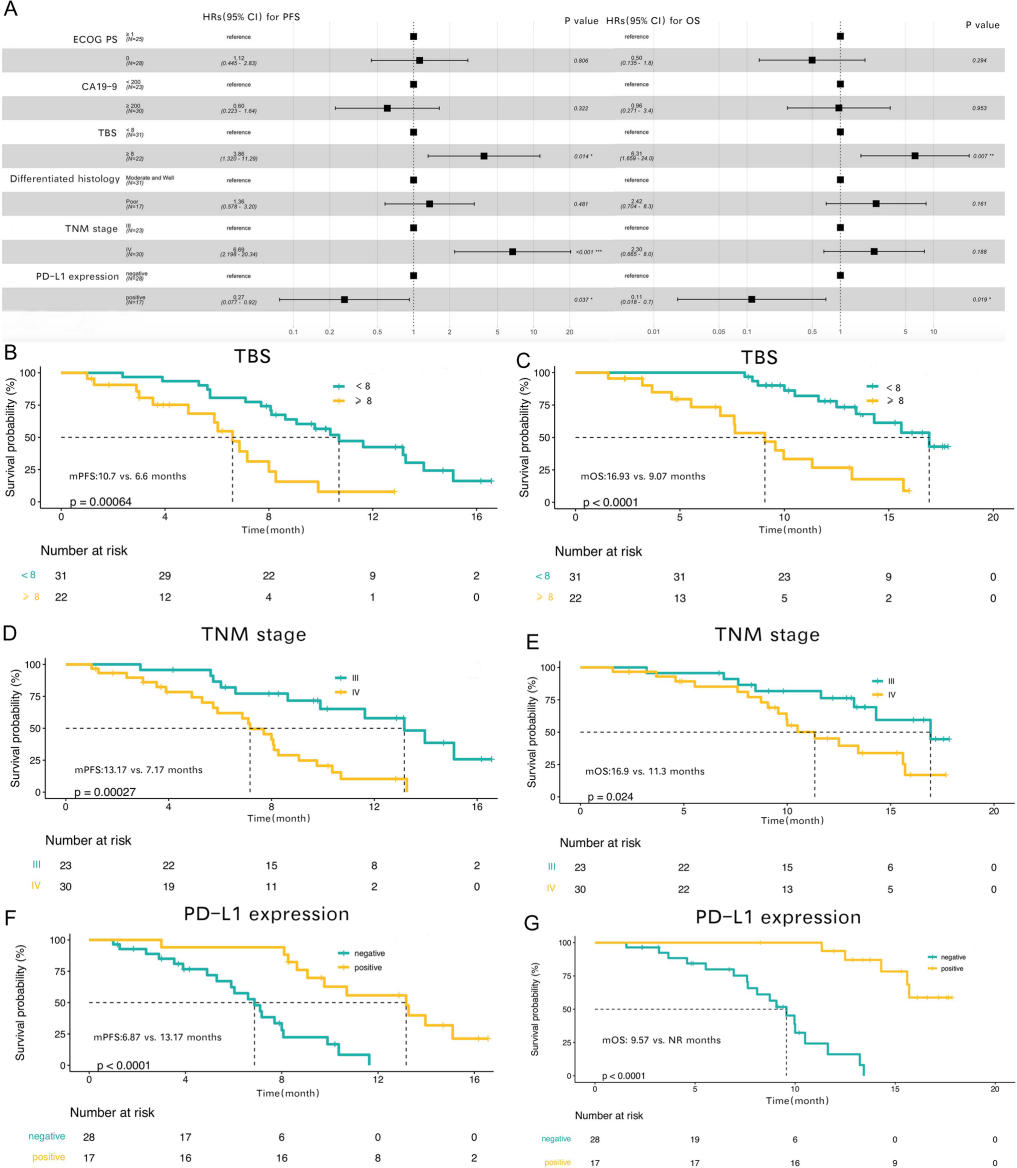
Subgroup analyses and prognostic factors. Subgroup analyses of progression-free survival (PFS) and overall survival (OS) in the entire cohort (**a**). Kaplan–Meier plots for PFS (**b**) and OS (**c**) based on TBS. Kaplan–Meier plots for PFS (**d**) and OS (**e**) based on the TNM stage. Kaplan–Meier plots for PFS (**f**) and OS (**g**) based on PD-L1 expression

Twelve potential prognostic variables for PFS and OS were first selected using univariate Cox analysis, including age, sex, ECOG PS, Child–Pugh score, CA19-9, CEA, HBV infection, maximum tumor diameter, TBS, differentiated histology, TNM stage, and PD-L1 expression (Table 3). In the univariate Cox analysis, TBS (≥ 8 vs. < 8; HR: 3.5; 95% CI: 1.6–7.4; P = 0.001), TNM stage (IV vs. III; HR: 4; 95% CI: 1.8–8.8; P < 0.001), and PD-L1 expression (positive vs. negative; HR: 0.18; 95% CI: 0.071–0.44; P < 0.001) were different for PFS, and ECOG PS (0 vs. ≥ 1; HR: 0.33; 95% CI: 0.15–0.74, P = 0.007), CA19-9 (≥ 200 vs. < 200; HR: 3.8; 95% CI: 1.6–8.9; P = 0.003), TBS (≥ 8 vs. < 8; HR: 4.8; 95% CI: 2.1–11; P < 0.001), differentiated histology (poor vs. moderate + well; HR: 2.8; 95% CI: 1.1–6.7; P = 0.024), TNM stage (IV vs. III; HR: 2.6; 95% CI: 1.1–6.1; P = 0.029), and PD-L1 expression (positive vs. negative; HR: 0.044; 95% CI: 0.0095–0.2; P < 0.001) were different for OS. Six factors that differed in the univariate Cox analysis, including ECOG PS, CA19-9, TBS, differentiated histology, TNM stage, and PD-L1 expression, were further subjected to multivariate Cox analysis. In the multivariate Cox analysis, TBS (≥ 8 vs. < 8; HR: 3.86; 95% CI: 1.32–11.29; P = 0.014), TNM stage (IV vs. III; HR: 6.69; 95% CI: 2.198–20.34; P < 0.001), and PD-L1 expression (positive vs. negative; HR: 0.27; 95% CI: 0.077–0.92; P = 0.037) were different for PFS, and TBS (≥ 8 vs. < 8; HR: 6.31; 95% CI: 1.659–24.0; P = 0.007) and PD-L1 expression (positive vs. negative; HR: 0.11; 95% CI: 0.018–0.7; P = 0.019) were different for OS (Table 3, Fig. 3a).

**Table 3:** Univariate and multivariate analyses of prognostic factors for progression-free survival (PFS) and overall survival (OS)

Variate	Univariate analysis for PFS		Multivariate analysis for PFS		Univariate analysis for OS		Multivariate analysis for OS	
	HR (95% CI)	P value	HR (95% CI)	P value	HR (95% CI)	P value	HR (95% CI)	P value
Age (≥ 60 vs. < 60)	0.64 (0.32–1.3)	0.221			0.8 (0.36–1.8)	0.597		
Sex (Male vs. Female)	0.89 (0.45–1.8)	0.744			0.67 (0.3–1.5)	0.331		
ECOG PS (0 vs. ≥ 1)	0.68 (0.35–1.3)	0.267	1.12 (0.445–2.83)	0.806	0.33 (0.15–0.74)	**0.007**	0.50 (0.135–1.8)	0.294
Child–Pugh score (B vs. A)	1.6 (0.77–3.3)	0.211			1.5 (0.69–3.5)	0.294		
CA19-9 (≥ 200 vs. < 200)	2 (0.99–4.1)	0.052	0.60 (0.223–1.64)	0.322	3.8 (1.6–8.9)	**0.003**	0.96 (0.271–3.4)	0.953
CEA (≥ 5 vs. < 5)	1.6 (0.79–3.1)	0.193			2.1 (0.94–4.6)	0.071		
HBV infection (Yes vs. No)	0.68 (0.26–1.8)	0.431			1 (0.38–2.7)	0.989		
Maximum tumor diameter (≥ 5 vs. < 5)	0.79 (0.4–1.6)	0.505			1 (0.45–2.2)	0.992		
TBS (≥ 8 vs. < 8)	3.5 (1.6–7.4)	0.001^*^	3.86 (1.32–11.29)	**0.014**	4.8 (2.1–11)	** < 0.001**	6.31 (1.659–24.0)	**0.007**
Differentiated histology (Poor vs. Moderate + Well)	1.6 (0.75–3.3)	0.235	1.36 (0.578–3.20)	0.481	2.8 (1.1–6.7)	**0.024**	2.42 (0.704–8.3)	0.161
TNM stage (IV vs. III)	4 (1.8–8.8)	** < 0.001**	6.69 (2.198–20.34)	** < 0.001**	2.6 (1.1–6.1)	**0.029**	2.30 (0.665–8.0)	0.188
PD-L1 expression (positive vs. negative)	0.18 (0.071–0.44)	** < 0.001**	0.27 (0.077–0.92)	**0.037**	0.044 (0.0095–0.2)	** < 0.001**	0.11 (0.018–0.7)	**0.019**

*ECOG* Eastern Cooperative Oncology Group, *CA19-9* carbohydrate antigen 19–9, *CEA* carcinoembryonic antigen, *HBV* hepatitis type B virus, *TBS* tumor burden score, *TNM* tumor node metastasis classification, *PD-L1* programmed cell death ligand 1

*The font is bolded to emphasize that the P value is less than 0.05

Subgroup analyses of PFS and OS for three potentially prognostic variables, including TBS, TNM stage, and PD-L1 expression, were performed in the entire cohort. When we stratified patients according to TBS, the Kaplan–Meier survival curve and log-rank test analysis demonstrated that patients with TBS < 8 had a longer median PFS (10.7 vs. 6.6 months, P < 0.001; Fig. 3b) and a longer median OS (16.93 vs. 9.07 months, P < 0.001; Fig. 3c) than those with TBS ≥ 8. When we stratified patients according to the TNM stage, the Kaplan–Meier survival curve and log-rank test analysis showed that patients with TNM stage III had a longer median PFS (13.17 vs. 7.17 months, P < 0.001; Fig. 3d) and a longer median OS (16.9 vs. 11.3 months, P = 0.024; Fig. 3e) than those with TNM stage IV. When we stratified patients according to PD-L1 expression, the Kaplan–Meier survival curve and log-rank test analysis revealed that patients with positive PD-L1 expression had a longer median PFS (13.17 vs. 6.87 months, P < 0.001; Fig. 3f) and a longer median OS (NR vs. 9.57 months, P < 0.001; Fig. 3g) than those with negative PD-L1 expression.

### Tolerability and safety

**Fig. 4:**
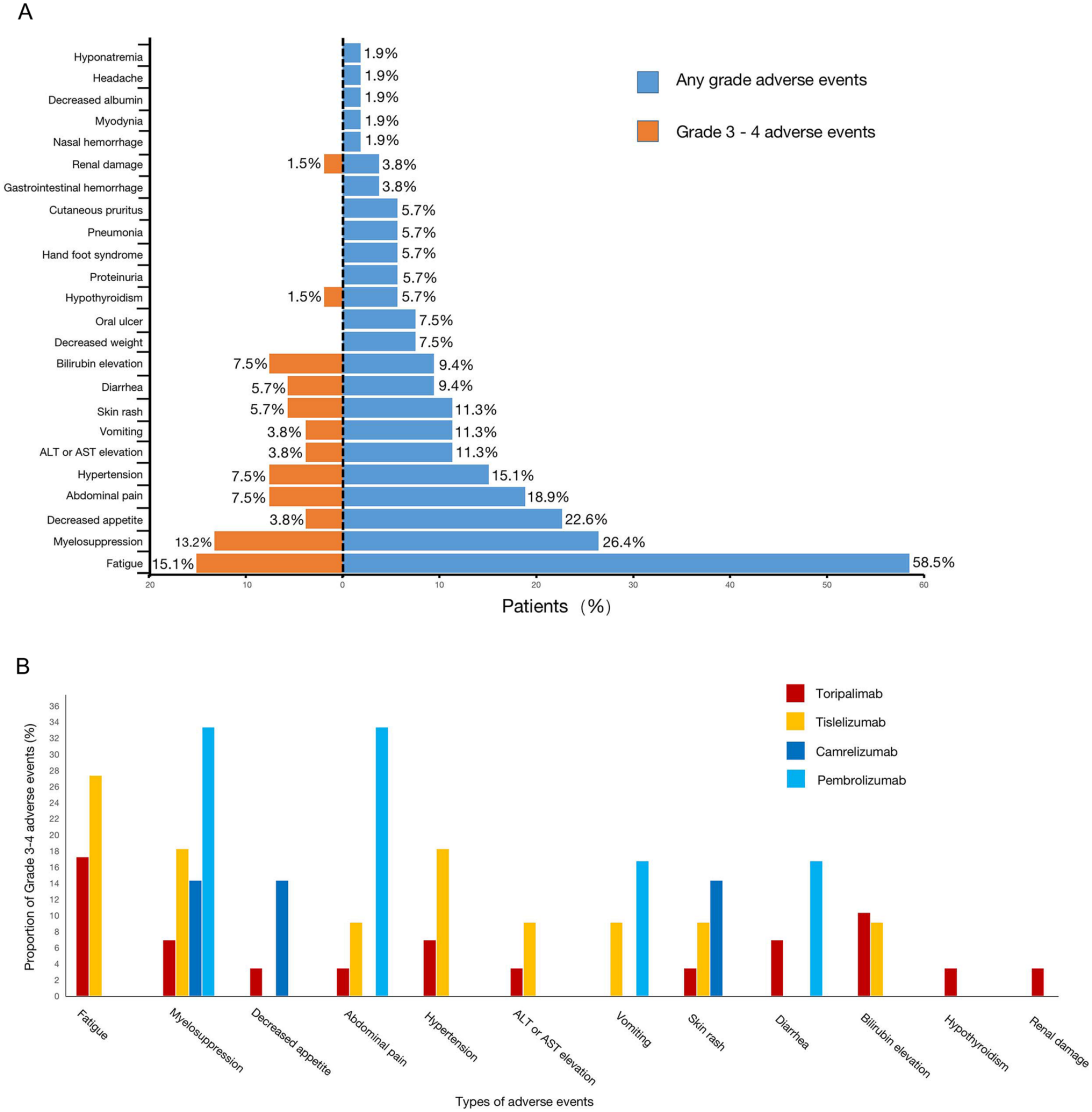
Frequency of any grade and grade 3/4 adverse events. Frequency of any grade and grade 3/4 adverse events (**a**). The proportion of grade 3/4 adverse events in the different PD-1 inhibitor groups (**b**)

AEs were reported in all 53 patients (100%) throughout the study (Table S4, Fig. 4a). However, we did not detect grade 5 AE. Regarding severe AEs (SAEs), 41.5% (22/53) of the patients had ≥ grade 3 AEs, and only 1.9% (1/53) experienced grade 4 AEs (myelosuppression). The most common AEs (of any grade) were fatigue (31/53, 58.5%), myelosuppression (14/53, 26.4%), and decreased appetite (12/53, 22.6%). Most AEs that occurred during combination immunotherapy were not fatal, well-tolerated, and controlled. Particularly, the most common grade 3 or 4 SAEs were fatigue (8/53, 15.1%), myelosuppression (7/53, 13.2%), abdominal pain (4/53, 7.5%), hypertension (4/53, 7.5%), and bilirubin elevation (4/53, 7.5%).

We compared the AEs that occurred in the different PD-1 inhibitor groups, and there was no statistically significant difference in grade 3–4 AEs between the different PD-1 inhibitor groups (Table S2, Fig. 4b). In the toripalimab group, the most common grade 3–4 AEs were fatigue (17.2%, 5/29) and bilirubin elevation (10.3%, 3/29). In the tislelizumab group, the most common grade 3–4 AEs were fatigue (27.3%, 3/11), myelosuppression (18.2%, 2/11), and hypertension (18.2%, 2/11). In the camrelizumab group, the most common grade 3–4 AEs were myelosuppression (14.3%, 1/7), decreased appetite (14.3%, 1/7), and skin rashes (14.3%, 1/7). In the pembrolizumab group, the most common grade 3–4 AEs were myelosuppression (33.3%, 2/6) and abdominal pain (33.3%, 2/6). After careful treatment, we discovered that all observed AEs were controllable.

## Discussion

To our knowledge, this is the first, largest sample size and multicenter study to investigate PD-1 inhibitors plus lenvatinib with Gemox chemotherapy as the first-line treatment option for advanced ICC in a real-world study. In this study, the triple combination regimens showed good efficacy and tolerable adverse reactions, with a median OS of 14.3, a median PFS of 8.63 months, and an ORR of 52.8%. Subgroup analysis confirmed three potential prognostic variables: TBS, TNM stage, and PD-L1 expression for PFS and OS. The rate of grade 3 and 4 AEs was 41.5% (22/53), which is acceptable, tolerable, and controllable.

PD-1 inhibitors, which are important components of ICIs, are increasingly used in BTC therapy [8, 24, 25]. Nivolumab combined with gemcitabine and tegafur chemotherapy has shown a good therapeutic effect in the first-line treatment of advanced BTC, with an ORR of 41.7% [24]. A study of PD-1 inhibitors plus lenvatinib for unresectable BTC showed an ORR of 42.1% [8]. These findings suggest that a combination of drugs with different mechanisms of action can overcome or improve the drug resistance of single-drug applications. Some studies suggest that chemotherapy may enhance the efficacy of PD-1 inhibitors through the following mechanisms: suppression of antitumor immunity by reducing myeloid-derived suppressor cells, selectively depleting monocytes/macrophages, enhancing the recruitment of antigen-presenting cells, and promoting the phagocytosis of dendritic cells through cytokines produced by cytotoxic chemotherapy damage to cancer cells [11–13]. Lenvatinib can promote the efficacy of immunotherapy by eliminating cancer cells through direct antitumor activity and immunogenic cell death and by reducing the number of cells targeted and destroyed by immune cells [26, 27].

Two clinical trials by Zhou et al. and Li et al. confirmed the efficacy of PD-1 inhibitors combined with lenvatinib and Gemox chemotherapy in ICC or BTC [16, 17]. When these two studies were compared with the current study, they consistently showed high ORRs despite using different PD-1 inhibitors and study endpoints (Table S3). The ORRs obtained in this study, the study by Zhou et al., and the study by Li were 52.8, 80, and 56%, respectively. The primary endpoint of the study by Li et al. was R0 resection rate (52%), with the major eligibility criteria being potentially resectable locally advanced BTC. In this study, 6 patients successfully underwent conversion surgery, suggesting that triple combined therapy regimens may be an option for patients with potential conversion surgery (Table S1, Figure S1). Multiple PD-1 inhibitors were used in our study compared to two reported clinical trials [16, 17]. Several other studies have reported that different types of PD-1 inhibitors have positive effects [8, 10, 22, 24, 25, 28]. We also performed subgroup analyses for different anti-PD-1 antibody regimens. We discovered that no significant differences were observed in the median PFS (9.90 vs. 7.55 vs. 7.62 vs. 9.77 months, P = 0.41; Figure S2A) and the median OS (11.6 vs. 13.5 vs. 11.3 vs. 15.6 months, P = 0.34; Figure S2B) among the camrelizumab, pembrolizumab, tislelizumab, and toripalimab groups. We performed subgroup analyses in the non-toripalimab and toripalimab groups and found no significant differences in the median PFS (8.0 vs. 9.77 months, P = 0.13; Figure S2C) and the median OS (11.6 vs. 15.6 months, P = 0.39, Figure S2D).

In this study, although each patient experienced varying degrees of AE, the incidence of severe AE was not significantly higher than that in other studies. Myelosuppression is a common AE of chemotherapy [3, 29]. In this study, 26.4% (14/53) of patients had varying degrees of myelosuppression, of which 11.3% (6/53) had grade 3–4 AE. In some studies on different combinations of PD-1 inhibitors, chemotherapy, and targeted therapy, the incidence of grade 3–4 AE was as high as 59.5% [3, 7, 29]. However, the incidence of grade 3–4 AE in our study was 41.5% (22/53), which is not higher than that reported in previous studies. In this study, no grade 5 AEs occurred, suggesting that PD-1 inhibitor plus lenvatinib with Gemox chemotherapy did not impose an additional burden on patients with AEs in the context of good efficacy.

This study has some limitations. First, although this was a multicenter real-world study, the total sample size was still limited due to the selection of treatment regimens and the incidence of diseases. In the future, multicenter cohort studies with larger sample sizes are needed to investigate the efficacy and tolerability of triple combined regimens. Second, multiple PD-1 inhibitors were administered in this study. Although there was no significant difference in survival and AE in the subgroup analysis, there may be a certain bias due to the small sample size of some PD-1 inhibitors. Future studies with single PD-1 inhibitors and large sample sizes are needed to verify whether there are differences among different PD-1 inhibitors. Third, this study lacks a cohort of standard chemotherapy-based regimens as controls, and prospective cohort study designs are needed to compensate for this deficiency in the future. Finally, although three potential prognostic variables were confirmed in this study, we were unable to collect and analyze more potential factors, such as the tumor mutational burden. Thus, future studies that include more prognostic factors should be conducted. Nonetheless, the results of this study can be used as a reference for the design of subsequent clinical studies and the selection of clinical treatment strategies.

In conclusion, PD-1 inhibitors combined with lenvatinib and Gemox chemotherapy are effective, safe, and well-tolerated as first-line therapies for advanced ICC. In addition, TBS, TNM stage, and PD-L1 expression can be used as potential prognostic factors.

## Supplementary Information

Figure S1. A patient with conversion surgery

Computed tomography (CT) image at pretreatment (A) and before surgery (B). H&E staining of the resected specimen (C). (D) Resected specimen.

Figure S2. Kaplan–Meier plot for progression-free survival (PFS) (A) and overall survival (OS) (B) based on four different types of PD-1 inhibitor groups. Kaplan–Meier plot for PFS (C) and OS (D) based on the non-toripalimab and toripalimab groups

Table S1. Main information of the six patients who underwent conversion surgery after triple combination treatment.

Table S2. Adverse events of different types of PD-1 inhibitors.

Table S3. The inclusion criteria, baseline characteristics, study endpoint, and therapeutic response of the present study compared with two other studies.

Table S4. Commonly observed adverse events. Below is the link to the electronic supplementary material.

Supplementary file1 (EPS 82497 KB)

Supplementary file2 (EPS 72010 KB)

Supplementary file3 (DOCX 26 KB)

Supplementary file4 (DOCX 18 KB)

## Data Availability

Data are available upon reasonable request.

## Abbreviations

AEs: Adverse events
BTC: Biliary tract cancers
CA19-9: Carbohydrate antigen 19-9
CEA: Carcinoembryonic antigen
CT: Computed tomography
CI: Confidence interval
CBR: Clinical benefit rate
CR: Complete response
DCR: Disease control rate
ECOG: Eastern Cooperative Oncology Group
FOLFOX: Folinic acid, fluorouracil, and oxaliplatin
Gemox: Oxaliplatin, and gemcitabine
GC: Gemcitabine plus cisplatin
HBV: Hepatitis B virus
HR: Hazard ratios
ICIs: Immune checkpoint inhibitors
ICC: Intrahepatic cholangiocarcinoma
MRI: Magnetic resonance imaging
OS: Overall survival
ORR: Objective response rate
PUMCH: Peking Union Medical College Hospital
PLAGH: The Fifth Medical Center of PLA General Hospital
PD-1: Programmed cell death protein-1
PFS: Progression-free survival
PR: Partial response
SD: Stable disease
TBS: Tumor burden score
TNM: Tumor-node metastasis classification
VEGFR: Vascular endothelial growth factor receptors
