# Supplementary material for: Efficacy, safety, and prognostic factors of PD-1 inhibitors combined with lenvatinib and Gemox chemotherapy as first-line treatment in advanced intrahepatic cholangiocarcinoma: a multicenter real-world study
Source: Cancer Immunol Immunother. 2023 May 29;72(9):2949–60. doi: 10.1007/s00262-023-03466-8 (PMC10412480; doi:10.1007/s00262-023-03466-8)
Supplement: Supplementary file 3 — Supplementary file3 (DOCX 26 KB) [file 262_2023_3466_MOESM3_ESM.docx]

**Supplementary Material**

**Table S1 Main information of the six patients underwent conversion surgery after triple combination treatment**

| Patient ID | Type of PD-1 inhibitors | Cycles of triple combination regimens | Duration treatment before conversion surgery (months) | Best therapeutic response assessment | Current disease status | Current treatment options |
| --- | --- | --- | --- | --- | --- | --- |
| 16 | Pembrolizumab | 6 | 13.9 | PR | Disease progression (1.2 months after conversion surgery) | Treatment changed after progression |
| 22 | Camrelizumab | 5 | 3.9 | PR | No disease progression | Camrelizumab + lenvatinib |
| 31 | Toripalimab | 5 | 3.8 | PR | No disease progression | Toripalimab + lenvatinib |
| 41 | Toripalimab | 5 | 4.17 | CR | No disease progression | Toripalimab + lenvatinib |
| 47 | Toripalimab | 2 | 1.03 | CR | No disease progression | Toripalimab + lenvatinib |
| 50 | Toripalimab | 4 | 3.3 | PR | No disease progression | Toripalimab + lenvatinib |

PR; partial response, CR; complete response

**Table S2 Adverse events of different types of PD-1 inhibitors**

|  | Toripalimab  (n=29) | | Tislelizumab  (n=11) | | Camrelizumab  (n=7) | | Pembrolizumab  (n=6) | |  |
| --- | --- | --- | --- | --- | --- | --- | --- | --- | --- |
| Adverse events | Any grade,  n (%) | Grade 3-4,  n (%) | Any grade,  n (%) | Grade 3-4,  n (%) | Any grade,  n (%) | Grade 3-4,  n (%) | Any grade,  n (%) | Grade 3-4,  n (%) | ^#^P value |
| Total | 29 (100) | 13 (44.8) | 11 (100) | 4 (36.3) | 7 (100) | 2 (28.6) | 6 (100) | 3 (50.0) | 0.854 |
| Fatigue | 20 (69.0) | 5 (17.2) | 6 (54.5) | 3 (27.3) | 3 (42.9) | 0 | 2 (33.3) | 0 | 0.383 |
| Myelosuppression | 4 (13.8) | 2 (6.9) | 5 (45.5) | 2 (18.2) | 2 (28.6) | 1 (14.3) | 3 (33.3) | 2 (33.3) | 0.172 |
| Decreased appetite | 4 (13.8) | 1 (3.4) | 3 (27.3) | 0 | 3 (42.9) | 1 (14.3) | 2 (50.0) | 0 | 0.474 |
| Abdominal pain | 3 (10.3) | 1 (3.4) | 2 (18.2) | 1 (9.1) | 1 (14.3) | 0 | 4 (66.7) | 2 (33.3) | 0.097 |
| Hypertension | 3 (10.3) | 2 (6.9) | 3 (27.3) | 2 (18.2) | 1 (14.3) | 0 | 1 (16.7) | 0 | 0.496 |
| ALT or AST elevation | 2 (6.9) | 1 (3.4) | 2 (18.2) | 1 (9.1) | 0 | 0 | 2 (33.3) | 0 | 0.705 |
| Vomiting | 0 | 0 | 2 (18.2) | 1 (9.1) | 1 (14.3) | 0 | 3 (33.3) | 1 (16.7) | 0.144 |
| Skin rash | 3 (10.3) | 1 (3.4) | 1 (9.1) | 1 (9.1) | 1 (14.3) | 1 (14.3) | 1 (16.7) | 0 | 0.428 |
| Diarrhea | 3 (10.3) | 2 (6.9) | 0 | 0 | 0 | 0 | 2 (33.3) | 1 (16.7) | 0.532 |
| Bilirubin elevation | 3 (10.3) | 3 (10.3) | 1 (9.1) | 1 (9.1) | 0 | 0 | 1 (16.7) | 0 | 1.000 |
| Decreased weight | 0 | 0 | 1 (9.1) | 0 | 1 (14.3) | 0 | 2 (33.3) | 0 | - |
| Oral ulcer | 1 (3.4) | 0 | 2 (18.2) | 0 | 1 (14.3) | 0 | 0 | 0 | - |
| Hypothyroidism | 1 (3.4) | 1 (3.4) | 1 (9.1) | 0 | 1 (14.3) | 0 | 0 | 0 | 1.000 |
| Proteinuria | 0 | 0 | 1 (9.1) | 0 | 1 (14.3) | 0 | 1 (16.7) | 0 | - |
| Hand foot syndrome | 0 | 0 | 1 (9.1) | 0 | 1 (14.3) | 0 | 1 (16.7) | 0 | - |
| Pneumonia | 0 | 0 | 2 (18.2) | 0 | 0 | 0 | 1 (16.7) | 0 | - |
| Cutaneous pruritus | 0 | 0 | 1 (9.1) | 0 | 2 (28.6) | 0 | 0 | 0 | - |
| Gastrointestinal hemorrhage | 0 | 0 | 1 (9.1) | 0 | 0 | 0 | 1 (16.7) | 0 | - |
| Renal damage | 2 (6.9) | 1 (3.4) | 0 | 0 | 0 | 0 | 0 | 0 | 1.000 |
| Nasal hemorrhage | 0 | 0 | 1 (9.1) | 0 | 0 | 0 | 0 | 0 | - |
| Myodynia | 1 (3.4) | 0 | 0 | 0 | 0 | 0 | 0 | 0 | - |
| Decreased albumin | 1 (3.4) | 0 | 0 | 0 | 0 | 0 | 0 | 0 | - |
| Headache | 1 (3.4) | 0 | 0 | 0 | 0 | 0 | 0 | 0 | - |
| Hyponatremia | 1 (3.4) | 0 | 0 | 0 | 0 | 0 | 0 | 0 | - |

^#^Grade 3-4 adverse event rates were compared among the 4 different types of PD-1 inhibitor groups

ALT; alanine aminotransferase, AST; aspartate aminotransferase

**Table S3 The inclusion criteria, baseline characteristics, study endpoint, and therapeutic response of present study compared with other two studies**

| Parameters | Present study (n = 53) | Study One by Zhou^#^ (n=30) | Study Two by Li* (n=25) |
| --- | --- | --- | --- |
| Inclusion criteria | Advanced ICC, no previous systemic treatment | Locally advanced or metastatic ICC, first-line treatment | Potentially resectable locally advanced BTC, no previous systemic treatment |
| Age, years (median, range) | 58 (31-74) | 56.5 (25-73） | 59.7 (33-77） |
| Sex, n [%] |  |  |  |
| Female | 20 [37.7] | 11 [37] | 14 [56] |
| Male | 33 [62.3] | 19 [63] | 11 [44] |
| ECOG PS, n [%] |  |  |  |
| 0 | 28 [52.8] | - | 25 [100] |
| 1 | 23 [43.4] | - | - |
| 2 | 2 [3.8] | - | - |
| Child–Pugh score, n [%] |  |  |  |
| A | 32 [60.4] | - | 23 [92] |
| B | 21 [39.6] | - | 2 [8] |
| Study endpoint |  |  |  |
| Primary endpoint | OS, PFS | ORR | R0 resection rate |
| Secondary endpoint | ORR, DCR, AE | PFS, OS | PFS, ORR, DCR |
| ORR (n, %, 95% CI) | 28, 52.8 (39.7-65.6) | 24, 80 (61.4%-92.3%) | 14, 56 (37.1-73.3) |
| CR (n, %) | 3 (5.7) | 1 (3.3) | 0 |
| PR (n, %) | 25 (47.1) | 23 (76.7) | 14 (56) |
| SD (n, %) | 22 (41.5) | 4 (13.3) | 9 (36) |
| PD (n, %) | 3 (5.7) | 2 (6.7) | 2 (8) |
| DCR (n, %, 95% CI) | 50, 94.3 (84.6-98.1) | 28, 93.3 (77.9-99.2) | 23, 92 (75.0-97.8) |
| CBR (n, %, 95% CI) | 40, 75.5 (62.4-85.1) | - | - |
| mPFS (months, 95% CI) | 8.63 (7.17–11.6) | 10.0 | - |
| mOS (months, 95% CI) | 14.3 (11.3–NE) | Not Reached | - |
| 12 months OS (%, 95% CI) | 59.6 (46.6-76.3) | 73.3 (57.5-89.2) | - |

^#^PD-1 inhibitor：Toripalimab；^*^PD-1 inhibitor：Tislelizumab

ICC; intrahepatic cholangiocarcinoma, BTC; biliary tract cancer, ECOG; Eastern Cooperative Oncology Group, OS; overall survival, PFS; progression free survival, ORR; objective response rate, DCR; disease control rate, AE; adverse events, CR; complete response, PR; partial response, SD; stable disease, PD; progressive disease, CBR; clinical benefit rate
