# Supplementary material for: Efficacy, safety, and prognostic factors of PD-1 inhibitors combined with lenvatinib and Gemox chemotherapy as first-line treatment in advanced intrahepatic cholangiocarcinoma: a multicenter real-world study
Source: Cancer Immunol Immunother. 2023 May 29;72(9):2949–60. doi: 10.1007/s00262-023-03466-8 (PMC10412480; doi:10.1007/s00262-023-03466-8)
Supplement: Supplementary file 4 — Supplementary file4 (DOCX 18 KB) [file 262_2023_3466_MOESM4_ESM.docx]

**Table 4 Commonly observed adverse events**

| Adverse events | Any grade, n (%) | Grade 3, n (%) | Grade 4, n (%) |
| --- | --- | --- | --- |
| Total | 53 (100) | 22 (41.5) | 1 (1.9) |
| Fatigue | 31 (58.5) | 8 (15.1) | 0 |
| Myelosuppression | 14 (26.4) | 6 (11.3) | 1 (1.9) |
| Decreased appetite | 12 (22.6) | 2 (3.8) | 0 |
| Abdominal pain | 10 (18.9) | 4 (7.5) | 0 |
| Hypertension | 8 (15.1) | 4 (7.5) | 0 |
| ALT or AST elevation | 6 (11.3) | 2 (3.8) | 0 |
| Vomiting | 6 (11.3) | 2 (3.8) | 0 |
| Skin rash | 6 (11.3) | 3 (5.7) | 0 |
| Diarrhea | 5 (9.4) | 3 (5.7) | 0 |
| Bilirubin elevation | 5 (9.4) | 4 (7.5) | 0 |
| Decreased weight | 4 (7.5) | 0 | 0 |
| Oral ulcer | 4 (7.5) | 0 | 0 |
| Hypothyroidism | 3 (5.7) | 1 (1.9) | 0 |
| Proteinuria | 3 (5.7) | 0 | 0 |
| Hand foot syndrome | 3 (5.7) | 0 | 0 |
| Pneumonia | 3 (5.7) | 0 | 0 |
| Cutaneous pruritus | 3 (5.7) | 0 | 0 |
| Gastrointestinal hemorrhage | 2 (3.8) | 0 | 0 |
| Renal damage | 2 (3.8) | 1 (1.9) | 0 |
| Nasal hemorrhage | 1 (1.9) | 0 | 0 |
| Myodynia | 1 (1.9) | 0 | 0 |
| Decreased albumin | 1 (1.9) | 0 | 0 |
| Headache | 1 (1.9) | 0 | 0 |
| Hyponatremia | 1 (1.9) | 0 | 0 |

ALT; alanine aminotransferase, AST; aspartate aminotransferase
